# ‘Lifts your spirits, lifts your mind’: A co‐produced mixed‐methods exploration of the benefits of green and blue spaces for mental wellbeing

**DOI:** 10.1111/hex.13773

**Published:** 2023-05-01

**Authors:** Claire McCartan, Gavin Davidson, Liam Bradley, Katherine Greer, Lee Knifton, Aodán Mulholland, Paul Webb, Chris White

**Affiliations:** ^1^ Regional Trauma Network, IMPACT Research Centre Northern Health and Social Care Trust Antrim UK; ^2^ School of Social Sciences, Education and Social Work Queen's University Belfast Belfast UK; ^3^ Praxis Care Belfast UK; ^4^ Mental Health Foundation Glasgow UK

**Keywords:** green and blue spaces, health inequalities, mental health, mixed methods, nature connectedness, physical health, wellbeing

## Abstract

**Introduction:**

Mental health problems are a considerable public health issue and spending time in nature has been promoted as a way to access a range of psychological benefits leading to the development of nature‐based interventions for people with severe and enduring mental health problems. Less, however, is understood about the potential benefits and efficacy of day‐to‐day routine access to outdoor green and blue spaces for mental health service users.

**Methods:**

Using a mixed‐methods design between April and October 2021, we explored the benefits and barriers to spending time outdoors with a purposive sample of mental health service users (*N* = 11) using qualitative interviews and an online general population survey (*N* = 1791). Qualitative evidence highlighted the restorative benefits of nature and identified a number of barriers associated with fears around personal safety, social anxiety, fatigue and lack of motivation. COVID‐19 had also restricted access to green and blue spaces. Having social contact and support encouraged people to spend time outdoors. In the quantitative survey, self‐report and standardised measures (the Patient Health Questionnaire and the Warwick–Edinburgh Wellbeing Scale) were used to assess past and current mental wellbeing.

**Findings:**

Statistically significant differences were found between wellbeing and the use of green and blue spaces. Those with mental health problems spent time outdoors because they: felt guilty; wanted to reduce their anxiety; or rely on someone for encouragement. Those without mental health problems endorsed more positively framed reasons including relaxation, improving physical health or getting exercise. Barriers for people with mental health problems involved safety concerns, feeling anxious and having a poor self‐image. These findings give insight into motivations for an outdoor activity to help inform the design of public mental health interventions.

**Conclusion:**

Further work is required to improve access and safety to promote the benefits of green and blue spaces for everyone.

**Patient or Public Contribution:**

The research team included expert experienced researchers with a mental health service provider (Praxis Care) and they were involved in the development of the research idea, funding application, design, data collection, analysis, writing up and dissemination activities.

## 1 INTRODUCTION

Spending time in nature has been connected to physical and mental health benefits and has led to the development of a wide range of nature‐based interventions designed to promote wellbeing, physical health and social inclusion for people with severe and enduring mental health problems.^1^ Disciplines, such as architecture, urban design, civil engineering and landscape architecture recognise the association between green and blue spaces and creating the physical and social context for people to live well. Mental health and addiction problems affect more than 1 billion people globally,^2^ and are estimated to cost over $6 trillion by 2030.^3^ People with mental health problems are at greater risk of developing life‐limiting health conditions such as cardiovascular disease, diabetes and obesity.^4^ Higher prevalence of risk‐taking health behaviours and lower levels of physical activity contribute to these risks.^5^, ^6^ Structural issues that reduce opportunities for health promotion and treatment disproportionately affect mental health service users.^7^, ^8^ Specialist living and care environments can also help reinforce some of these negative health behaviours and do not adequately promote healthy lifestyle changes.^9^, ^10^, ^11^ ‘Green’ spaces are urban or rural settings with natural vegetation, for example, woodland/forest, open countryside or city landscaping such as parkland, city trees, gardens or allotments; ‘blue’ spaces are characteristic of natural surface water including lakes, rivers or coastal waters but can include constructed urban waterways, canals or ponds.

Spending time outdoors can convey health and wellbeing benefits through a variety of means. Natural environments provide interest and offer opportunities to escape from daily hassles or worries^12^ and provide a contrast to overstimulating urban environments that are less restorative.^13^ Typically, outdoor space is used for physical activity and the mental health benefits of exercise have been well documented.^14^, ^15^ Even low levels of physical activity have the potential to improve cardiovascular health,^16^, ^17^ increase bone and muscle strength, improve sleep^18^ and generate feelings of wellbeing.^19^, ^20^ Physical activity can help improve self‐esteem which in turn is associated with healthy lifestyle behaviours.^21^, ^22^

Being outdoors creates opportunities to establish social networks and increase social capital that can contribute to wellbeing,^23^, ^24^, ^25^, ^26^ improve neighbourhood social cohesion and harness community engagement.^27^, ^28^, ^29^ Associations between natural vegetation and lower crime rates have also been observed.^30^, ^31^, ^32^ The economic benefits have also been researched. Saraev et al. estimated that visits to the UK's woodlands have helped cut costs associated with anxiety and depression by £185m (in 2020 prices) through a reduction in general practitioner (GP) visits, prescriptions, inpatient care and social services use.^33^

Although the evidence base is limited,^34^ access to light, fresh air and views of nature may increase wellbeing, job satisfaction, concentration and cognitive performance, and lower stress and depression.^12^, ^13^, ^24^, ^35^, ^36^, ^37^, ^38^ Good architecture acknowledges this established link by creating access to natural light and ventilation, forming landscapes and vistas to promote wellbeing and by limiting the use of toxic construction materials. Maximising the use of natural materials, and indoor and outdoor planting schemes create connections to the outside.^39^, ^40^ In healthcare settings, patients recovering from illness achieve better psychological wellbeing and support for their recovery when able to access the outdoors.^41^, ^42^, ^43^

Even *perceptions* of one's health^44^ and satisfaction with life may be improved by spending time outdoors.^45^, ^46^, ^47^ Brief exposure can have beneficial effects.^48^, ^49^, ^50^ As part of a study by the University of Essex,^48^ the authors recommended that ecotherapy should be a clinically recognised prescription treatment for mental distress; care planning should consider access to green space and access to green space should be a human right. National population data in Denmark found an association between a lack of access to green and blue spaces and up to a 55% chance of developing a psychological disorder.^50^ People living in more deprived settings typically have less access to green and blue spaces.^51^

While the many benefits of green and blue spaces have been explored, it is also possible for individuals to experience negative associations with green and blue spaces. Nature can evoke overwhelming, existential anxieties: climate change, the ruthlessness of survival within nature and the ‘perspective‐making power of nature,^52^ ^,^^p.376^ can lead people to reflect on their life, and ‘one's priorities and possibilities, on one's actions and one's goals’^53^ ^,^^p.197^. Spending time in nature can compound feelings of isolation or be a reminder of how disconnected everyday life can be from the physical world.^54^

We know less about the benefits and effectiveness of routine access to green and blue spaces for mental health users. To explore these issues further, we used a mixed‐methods design to identify potential barriers to spending time outdoors with a purposive sample of mental health service users (*N* = 11) and a general population survey accessed online (*N* = 1791) (April–October 2021).

## 2 METHODS

We used a co‐produced sequential mixed‐methods design. Thematic analysis of qualitative semistructured interviews, along with evidence from the research literature, was used to inform a cross‐sectional online survey to answer two questions:

1. How do people experiencing mental health problems use and benefit from green and blue spaces?
2. And are there differences in the frequency and use of green and blue spaces between people with and without mental health problems?

### 2.1 Impact of COVID‐19

The original design involved walking interviews however COVID‐19 restrictions meant face‐to‐face contact with research participants was prohibited. Telephone interviews were conducted instead. Access to the Closing the Gap: Health and Wellbeing Cohort to sample a population with experience of a severe mental illness (SMI) was no longer possible due to working‐from‐home restrictions to ameliorate this, we distributed paper questionnaires within Praxis Care's supported living settings.

### 2.2 Participants

Participants for the qualitative interviews were in receipt of a range of different services (e.g., supported living, day activities and befriending) and were invited to participate by the Head of Research in Praxis Care based in Northern Ireland.

Participants for the quantitative online survey were recruited through a number of different sources in the United Kingdom and the United States including a targeted sample of people interested in mental health issues accessed through Praxis Care's internal communication network and via the Mental Health Foundation and Praxis Care social media platforms (Twitter and Facebook), and a general population sample using Amazon MTurk's survey tool.

### 2.3 Qualitative data collection and analysis

A semistructured interview framework was developed to explore a range of themes with a purposive sample of mental health service users recruited through Praxis Care services. The qualitative interviews explored: exposure, frequency and dose of accessing green and blue spaces—time of day, social connections; facilitators and barriers to accessing green and blue spaces; benefits; negative effects and past experiences and future hopes.

Telephone interviews were conducted with 11 participants between February and April 2021. Three members of the research team, including two experts by experience, carried out one‐to‐one interviews lasting approximately 45 min each. Qualitative interviews were digitally recorded and transcribed for analysis by P. W. Thematic analysis^55^ was conducted by C. M., the key themes were reviewed, discussed and agreed upon with four members of the research team (including the experts by experience conducting the qualitative interviews) using the six‐phase process described by Braun and Clarke.^55^, ^56^ This stepped approach involved (1) *data familiarisation and writing familiarisation notes*; (2) *systematic data coding*; (3) *generating initial themes from coded and collated data*; (4) *developing and reviewing themes*; (5) *refining, defining and naming themes*; (6) *writing the report*.

### 2.4 Quantitative measures

Findings from the qualitative interviews and the research literature were used to develop a quantitative online survey using the Qualtrics™ platform. The short online survey was advertised online and included the following measures.

### 2.5 Demographics

Demographic information: year of birth; gender identity; sexual identity; disability status; relationship status; dependents and carer status; employment status; household financial coping; ethnicity; current or previous mental health diagnosis; current medication; city/country location.

### 2.6 Mental health problems

Experience of mental health problems was assessed using a self‐report measure, endorsement of any of the following statements was coded as a dichotomous variable ‘Yes’ (‘Yes, I have a current mental health problem diagnosed in the past 12 months’; ‘Yes, I have a current mental health problem that was diagnosed MORE than 12 months ago’; ‘Yes, I have been diagnosed in the past, but not currently experiencing any problems’ or ‘Yes, I have problems with my mental health but have never been diagnosed by a doctor‘) or ‘No’: ‘I have no mental health problems’.

### 2.7 Patient Health Questionnaire (PHQ‐8)

The PHQ‐8 is a well‐validated self‐report measure for assessing depressive symptom severity^57^ demonstrating good internal consistency reliability (Cronbach's *α* = .82–.85).^58^, ^59^ It asks respondents to report ‘Over the last 2 weeks, how often have you been bothered by…’ mood, sleep, energy levels, appetite, self‐esteem, and concentration. Based on the 9‐item measure (PHQ‐9), the PHQ‐8 omits the suicide risk assessment. This question was excluded for ethical reasons because appropriate follow‐up support could not be provided in our anonymised survey. The omission of the suicide risk item does not affect the reliability or validity of the measure.^60^ Total scores were calculated by assigning counts to the response categories of ‘not at all’ (0), ‘several days’ (1), ‘more than half the days’ (2), and ‘nearly every day’ (3). A cut‐off of 10 or greater was used to indicate a possible clinically significant level of depressive symptoms.

### 2.8 Warwick–Edinburgh Mental Wellbeing Scale

The short version of the Warwick–Edinburgh Mental Wellbeing Scale (SWEMWBS)^61^ was used to measure wellbeing. The SWEMWBS comprises seven positively worded statements rated on a 5‐point Likert scale and has been validated widely in different countries and age groups. The internal consistency reliability is high (Cronbach's *α* = .89–.93).^62^, ^63^, ^64^ Participants were asked to reflect on the previous 2 weeks and indicate to what degree they agreed with the statements. Sample items include: ‘I've been feeling optimistic about the future’; and ‘I've been able to make up my own mind about things’. The scale represents a score for each item from 1 to 5, where 1 = none of the time, and 5 = all of the time. All items are scored positively; a higher score indicates a higher level of mental well‐being. Total scores were used to indicate the current level of wellbeing: low (14–42); medium (43–60) and high (61–70).

### 2.9 Frequency, time of day and reasons for spending time outdoors

Participants were asked ‘over the past month, on average, how often have you spent time outdoors?’ and could choose one selection from seven items: ‘more than once a day’, ‘once a day’, ‘several times a week’, ‘once a week’, ‘once every 2 weeks’, ‘once’, ‘none’. This applied to any free or leisure time not spent working, job hunting, studying or doing household chores. They were then asked ‘Is there a time of day you prefer?’ A range of options was available from early morning to night time, participants could endorse multiple items.

### 2.10 Facilitators and barriers to green and blue spaces

Questions about facilitators and barriers were generated from the research literature. Participants were asked to indicate ‘what encourages you to spend time outdoors? ‘the weather’; ‘my mood’; ‘my friends/family’; ‘the right clothing/shoes’; ‘conservation/environmental reasons’; ‘to get a break from other people’; ‘my pets’ and a free text option of ‘other’. Respondents were asked ‘Not everyone enjoys spending time outdoors, are there times when you don't like going outside?’ and could endorse as many items that applied to them from: ‘no free time/other commitments’; ‘feeling unsafe’; ‘it's difficult to get to/access’; ‘the weather’; ‘how I look/feel about myself’ and ‘makes me feel anxious’.

## 3 FINDINGS

### 3.1 Qualitative findings

#### 3.1.1 Participants

Eleven service users (six women and five men) participated in the telephone semistructured interviews; each interview lasted around 45 min. They ranged in age from 34 to 84 years old (*M* = 53, SD = 12.61). The use of Praxis Care services was a proxy measure of an existing mental health problem that could range from mild‐moderate to severe and enduring.

#### 3.1.2 Type, frequency and dose

The majority of participants spent time outdoors every day, ranging from half an hour to 4 h a day. Tasks or errands associated with going outside provided motivation, something that helped provide structure to the day, ‘I get some exercise, go with a purpose, a job to do, an errand to run‥’ (Male, 40 years). For others who viewed leaving the house for everyday tasks as a source of anxiety, ‘I do like to go out but it's just that I get butterflies…you panic a bit and then you want to go back to the house…’ (Male, 47 years).

#### 3.1.3 Nature as restorative

Interviewees made a clear association between spending time outdoors and mental wellbeing. The word ‘lift’ was used to describe the effects of spending time in green and blue spaces, ‘lift your spirits’ (Female, 84 years), ‘lifts your mind’ (Female, 55 years) and ‘the psychological benefits are it keeps my mood lifted’ (Female, 50 years). Nature provided a contrast to the built environment, where ‘concrete almost wears you out…nature… it's uplifting you’ (Female 1, 47 years).

Enjoying wildlife, specifically birds and birdsong, was mentioned by half of those interviewed, and functioned as a way of connecting with nature.

#### 3.1.4 Pure and cleansing elements of nature

Nature was described as calming, soothing, and peaceful and expressed as pure and cleansing, ‘I think it clears you and you are sharper, yes, that's the purification thing, it just gives; it's so inspiring’ (Female 1, 47 years). Negative elements of the outdoors were also referred to as a source of anxiety including litter, dog fouling, rats and chewing gum on the pavements.

#### 3.1.5 Fear and safety

Fear and safety issues were raised in a number of different contexts. Practical safety issues were a concern for some. Personal safety, traffic, lack of footpaths or fear of falling and injury was also a concern, ‘I get anxious over my physical issues because I'm scared of falling’ (Female, 55 years). Antisocial behaviour, such as alcohol and drug use in public spaces, was also considered a barrier.

Fear relating to social anxiety and experiencing panic attacks was expressed by a number of respondents trying to manage their negative feelings.

#### 3.1.6 Social aspect

The opportunity that public spaces provide to promote social cohesion also resonated with half of the respondents talking about the social aspect of spending time outdoors, whether this was ‘talking and walking’ (Male, 60 years) or having the opportunity to ‘get to meet people’ (Male, 34 years) and ‘say hello, there is a kind of little community, social thing’ (Female 2, 47 years).

#### 3.1.7 Impact of COVID‐19

Many of those interviewed were vulnerable and became increasingly isolated during the lockdown. Some people relied on others to spend time outdoors, whether this was a family member or friend or the support services that Praxis Care offered. When these services were withdrawn, this impacted considerably their capacity to leave their home. ‘Now, we can't meet people. People are important to other people. They help each other’ (Male, 40 years).

#### 3.1.8 Barriers

A number of barriers to spending time in green and blue spaces were discussed. Lack of transport was an issue, especially for those living in rural areas. Physical health and tiredness could be a limiting factor, but problems associated with social isolation impacted being outdoors, ‘It's just not a place I would go to on my own, you know, I wouldn't ever think of going to a park’ because you would need company to go there (Female, 55 years). Mental health difficulties also could restrict attempts to socialise or leave the home, ‘I went and nobody talked to me and then I got really down so that's why I don't go out much’ (Male, 47 years).

#### 3.1.9 Facilitators

All of the interviewers were connected to Praxis Care, either as service users or employees, and reference was made to the support the organisation offered people, from providing social contact over the telephone, ‘you know when you get the phone call, it makes you feel better, makes you think that someone is thinking about you “cause you get lonely on your own”’ (Male, 47 years) to calling to the house and encouraging them to get some exercise and fresh air, ‘Praxis Care, the wee girls came with me for a walk and it makes me feel good…we go for a walk round the park and we talk, and then come back to the house, it makes me feel out of breath’ (Male, 47 years). Other factors that contributed to spending time outdoors were associated with promoting mental wellbeing, undertaking functional household‐related activities (shopping, medication, chores) and the opportunity for social contact.

### 3.2 Quantitative findings

#### 3.2.1 The sample

Between September and November 2021, a total of *N* = 1791 people participated in the online survey. The demographics of the sample are summarised in Table 1.

**Table 1:** Demographics.

Demographics	Total participants, *n* = (%)	Any mental health problems, *n* = (%)	No mental health problems, *n* = (%)
Gender			
Female	925 (52.4)	457 (25.9)*	468 (26.5)
Male	839 (47.6)	332 (18.8)	507 (28.7)
Total	1764 (100.0)	802 (44.9)	984 (55.1)
Ethnicity			
White	1438 (80.7)	677 (38.0)	761 (42.7)
Asian	104 (5.8)	34 (1.9)	70 (3.9)
Black	138 (7.7)	44 (2.5)	94 (5.3)
Hispanic	71 (4.0)	32 (1.8)	39 (2.2)
Other	30 (1.7)	14 (0.8)	16 (0.9)
Total	1781 (100.0)	801 (45.0)	980 (55.0)
Age			
18–39	869 (48.5)	428 (23.9)	441 (24.6)
40–59	412 (23.0)	194 (10.8)	218 (12.2)
60+	431 (24.1)	150 (8.4)	281 (15.7)
Total	1791 (100.0)	803 (44.8)	988 (55.2)
Relationship status			
Single	734 (41.4)	337 (19.0)	397 (22.4)
With a partner	1040 (58.6)	463 (26.1)	577 (32.5)
Total	1774 (100.0)	800 (45.1)	974 (54.9)
Sexual orientation			
Heterosexual or straight	1541 (88.6)	641 (41.6)	900 (58.4)
Gay or lesbian	38 (2.2)	28 (1.6)*	10 (0.6)
Bisexual	161 (9.3)	114 (6.6)	47 (2.7)
Total	1740 (100.0)	783 (45.0)	957 (55.0)
Disability			
Any disability	803 (44.8)	405 (22.6)*	398 (22.2)
2+ disabilities	114 (6.4)	99 (5.5)	15 (0.8)
No disabilities	988 (55.2)	150 (8.4)	838 (46.8)
Total	1791 (100.0)	555 (31.0)	1236 (69.0)

*Note*: χ^2^ test of independence.

Abbreviation: PHQ, patient health questionnaire.

*p <.005

#### 3.2.2 Frequency, time of day and reasons for accessing green and blue spaces

The majority of participants had access to green (90.8%) and blue spaces (69.6%) (Table 2). People with no experience of mental health problems reported more frequent use of green and blue spaces, at least once or more than once a day compared to those with experience of mental health problems. A significant relationship was observed between self‐reported mental health problems (*χ* ^2^(6, *N* = 1704) = 23.57, *p* = .001) or current wellbeing using the PHQ‐8 cutoff score of 10 or more (*χ* ^2^(6, *N* = 1652) = 61.25, *p* < .000). Respondents scoring ‘high’ on the SWEMWBS scale were also more likely than those rated ‘low’ or ‘medium’ to spend time outdoors at least once or more than once a day.

**Table 2:** Frequency of accessing green and blue spaces.

Frequency	No mental health problems, *n* = 919 (53.9%)	Any mental health problems, *n* = 785 (46.1%)	PHQ < 10, *n* = 1272 (77.0%)	PHQ 10+, *n* = 380 (23.0%)
More than once a day	203 (22.1)	130 (16.6)	277 (21.8)	44 (11.6)
Once a day	219 (23.8)	164 (20.9)	297 (23.3)	72 (18.9)
Several times a week	310 (33.7)	264 (33.6)	439 (34.5)	120 (31.6)
Once a week	109 (11.9)	133 (16.9)	159 (12.5)	76 (20.0)
Once every 2 weeks	45 (4.9)	43 (5.5)	49 (3.9)	37 (9.7)
Once	17 (1.8)	28 (3.6)	30 (2.4)	15 (3.9)
None	16 (1.7)	23 (2.9)	21 (1.7)	16 (4.2)

Abbreviation: PHQ, Patient Health Questionnaire.

Asked to consider their activity over the past month, respondents endorsed the different reasons why they had spent time outdoors (Table 3). Again, statistically significant differences were reported between those with, and those without, mental health problems. Participants disclosing mental health problems were more likely to endorse psychosocial‐related reasons for being outdoors including: ‘to let off steam’; because I feel guilty when I stay indoors'; ‘to reduce my anxiety’ and ‘to help me sleep’. In contrast, those with no experience of mental health problems cited more positively framed reasons for being outdoors including ‘to relax/unwind’; ‘to get some exercise/physical activity’; ‘to enjoy the weather’; ‘to improve my physical health’ and ‘working outdoors e.g. gardening’. Using the PHQ‐8 as a measure of current wellbeing showed similar trends. Those reporting possible clinical levels of depressive symptoms were more likely to have spent time outdoors because of external motivations such as walking a pet or because someone encouraged them to. Respondents scoring less than 10 on the PHQ‐8 endorsed a larger range of items including the positively framed reasons identified above as well as: ‘to get fresh air’; ‘have some time to myself’; ‘to improve my mood/feel better’; ‘to go shopping’ and ‘visit a friend or family member’. Greater variation in responses in the under 10 scores may reflect the inclusion of a subclinical sample with mild depressive symptoms scoring between 5 and 9 on the PHQ‐8.

**Table 3:** Over the past month, why have you spent time outdoors?

Reason	No mental health problems, *n* = 919 (53.9%)	Any mental health problems, *n* = 785 (46.1%)	PHQ < 10, *n* = 1272 (77.0%)	PHQ 10+, *n* = 380 (23.0%)
To relax/unwind	586 (59.3)***	422 (52.6)	841 (65.6)***	137 (36.0)
To walk my pet	303 (30.7)	256 (31.9)	394 (30.7)	147 (38.6)**
To meet up with friends/family	270 (27.3)	206 (25.7)	352 (27.5)	107 (28.1)
To get fresh air	634 (64.2)	516 (64.3)	923 (72.0)***	197 (51.7)
To get some exercise/physical activity	582 (58.9)***	394 (49.1)	813 (63.4)***	131 (34.4)
Someone encourages me to	38 (3.9)	56 (7.0)**	57 (4.5)	37 (9.7)***
To let off steam	96 (9.7)	119 (14.8)**	158 (12.3)	50 (13.1)
To travel to work, school or college (e.g., commuting by walking or cycling)	101 (10.2)	88 (11.0)	150 (11.7)	34 (8.9)
To enjoy nature/the environment	435 (44.0)	335 (41.7)	632 (49.3)***	113 (29.7)
Have some time to myself	283 (28.6)	237 (29.5)	410 (32.0)**	91 (23.9)
To improve my mood/feel better	329 (33.3)	310 (38.6)	497 (38.8)*	120 (31.5)
To help my concentration	80 (8.1)	76 (9.5)	120 (9.4)	30 (7.9)
To enjoy the weather	509 (51.5)**	380 (47.3)	746 (58.2)***	121 (31.8)
Because I feel guilty when I stay indoors	71 (7.2)	119 (14.8)***	136 (10.6)	51 (13.4)
To improve my mental health	156 (15.8)	264 (32.9)***	311 (24.3)	94 (24.7)
To help me think through or work out a problem	81 (8.2)	81 (10.1)	115 (9.0)	40 (10.5)
To help my motivation	95 (9.6)	95 (11.8)	143 (11.2)	37 (9.7)
To reduce my anxiety	114 (11.5)	215 (26.8)***	232 (18.1)	86 (22.6)
To help me sleep	68 (6.9)	80 (10.0)*	99 (7.7)	39 (10.2)
To improve my physical health	334 (33.8)**	224 (27.9)	476 (37.1)***	67 (17.6)
To lose weight	139 (14.1)	131 (16.3)	209 (16.3)	53 (13.9)
To improve my physical appearance	113 (11.4)	103 (12.8)	165 (12.9)	45 (11.8)
To go shopping	261 (26.4)	217 (27.0)	379 (24.9)*	87 (22.8)
To take part in a planned activity e.g. walking group, litter picking, outdoor gym	89 (9.0)	77 (9.6)	128 (10.0)	33 (8.7)
Visit a friend or family member	152 (15.4)	115 (14.3)	213 (16.6)**	40 (10.5)
Go to the library/doctor's surgery/place of worship	76 (7.7)	72 (9.0)	117 (9.1)	26 (6.8)
Working outdoors e.g. gardening	253 (25.6)***	146 (18.2)	348 (27.2)***	43 (11.3)

*Note*: *χ* ^2^ test of independence.

Abbreviation: PHQ, Patient Health Questionnaire.

* *p* < .05

** *p* < .005

*** *p* < .000.

Participants were also asked if there was a particular time of day that they preferred to go outdoors, multiple items could be endorsed (Table 4). Again significant differences were observed. Those with experience of mental health problems were more likely to endorse lunchtime (*χ* ^2^(1, *N* = 1791) = 5.36, *p* = .021) or night time *χ* ^2^(1, *N* = 1791) = 14.06, *p* < .000). Night‐time preference was also observed in the PHQ‐8 > 10 samples (*χ* ^2^(1, *N* = 1663) = 4.18, *p* = .041). A significant relationship was observed between respondents scoring ‘high’ levels of wellbeing on the SWEMWBS and spending time outdoors in the early morning (χ^2^(2, *N* = 1643) = 18.98, *p* < .000).

**Table 4:** Preferred time of day.

Time of day	No mental health problems, *n* = 988 (%)	Any mental health problems, *n* = 803 (%)	PHQ < 10, *n* = 1282 (%)	PHQ 10+, *n* = 381 (%)
Early morning	325 (32.9)	273 (34.0)	457 (35.6)	126 (33.1)
Midmorning	270 (27.3)	219 (27.3)	372 (29.0)	100 (26.2)
Lunchtime	151 (15.3)	156 (19.4)*	227 (17.7)	75 (19.7)
Midafternoon	299 (30.3)	260 (32.4)	450 (35.1)***	95 (24.9)
Early evening	474 (48.0)	418 (52.1)	693 (54.1)*	180 (47.2)
Night time	135 (13.7)	163 (20.3)**	79 (6.2)	287 (75.3)*
No preference	128 (13.0)	95 (11.8)	179 (14.0)**	32 (8.4)

*Note*: *χ* ^2^ test of independence.

Abbreviation: PHQ, Patient Health Questionnaire.

* *p* < .05

** *p* < .005

*** *p* < .000.

#### 3.2.3 Facilitators and barriers to accessing Green and blue spaces

What encourages you to spend time outdoors?: Facilitators for those with experience of any mental health problems included ‘my mood’, ‘the right clothing/shoes’ and ‘to get a break from other people’. For those without self‐reported mental health problems were more likely to be encouraged by the weather to go outdoors than those with experience of problems (*χ* ^2^(1, *N* = 1791) = 5.85, *p* = .016) (Table 5). The weather was also statistically significant for those scoring under 10 in the PHQ‐8, along with ‘to get a break from other people’ and ‘my pets’. Only those in the high wellbeing category reached statistical significance for ‘conservation/environmental reasons’ (*χ* ^2^(2, *N* = 1643) = 18.91, *p* < .000). *N* = 125 respondents provided an answer to the ‘other’ open‐ended question and exercise (*n* = 33) was the most popular reason for spending time outdoors, spending time in nature (*n* = 16) and gardening (*n* = 11).

**Table 5:** What encourages you to spend time outdoors?

Reason	No mental health problems, *n* = 988 (53.9%)	Any mental health problems, *n* = 803 (46.1%)	PHQ < 10, *n* = 1272 (77.0%)	PHQ 10+, *n* = 380 (23.0%)
The weather	772 (78.1)*	588 (73.2)	1105 (86.2)***	221 (58.0)
My mood	546 (55.3)	514 (64.0)***	801 (62.5)	230 (60.4)
My friends/family	408 (41.3)	335 (41.7)	549 (42.8)	172 (45.1)
The right clothing/shoes	108 (10.9)	121 (15.1)**	176 (13.7)	46 (12.1)
Conservation/environmental reasons	129 (13.1)	96 (12.0)	171 (13.3)	47 (12.3)
To get a break from other people	268 (27.1)	264 (32.9)**	418 (32.6)*	103 (27.0)
My pets	270 (27.3)	222 (27.6)	387 (30.2)**	87 (22.8)

*Note*: *χ* ^2^ test of independence.

Abbreviation: PHQ, Patient Health Questionnaire.

* *p* < .05

** *p* < .005

*** *p* < .000.

Not everyone enjoys spending time outdoors, are there times when you don't like going outside?: Participants with experience of past or current problems were statistically significantly more likely to identify barriers such as ‘feeling unsafe’, ‘how I look/feel about myself’ and ‘makes me feel anxious’ than those without mental health problems (Table 6). They were also more likely to find it ‘difficult to get access’. Significant associations were observed for those scoring less than 10 on the PHQ‐8 for barriers relating to ‘no free time/other commitments’ and ‘the weather’ compared to those reporting mental health problems. The open‐ended ‘other’ response generated *N* = 59 responses. The most frequently cited reason was health limitations (*n* = 12), and insects/bugs (*n* = 10).

**Table 6:** Are there any times when you don't like going outside?

Barriers	No mental health problems, *n* = 988 (53.9%)	Any mental health problems, *n* = 803 (46.1%)	PHQ < 10, *n* = 1272 (77.0%)	PHQ 10+, *n* = 380 (23.0%)
No free time/other commitments	328 (33.2)	276 (34.4)	481 (37.5)**	106 (27.8)
Feeling unsafe	137 (13.9)	201 (25.0)***	206 (16.1)	127 (33.3)***
It's difficult to get to/access	58 (5.9)	92 (11.5)***	79 (6.2)	67 (17.6)***
The weather	681 (68.9)	561 (69.9)	968 (75.5)***	243 (63.8)
How I look/feel about myself	76 (7.7)	178 (22.2)***	134 (10.5)	114 (29.9)***
Makes me feel anxious	28 (2.8)	130 (16.2)***	81 (6.3)	75 (19.7)***

*Note*: *χ* ^2^ test of independence.

Abbreviation: PHQ, Patient Health Questionnaire.

** *p* < .005

*** *p* < .000.

## 4 DISCUSSION

Spending time outdoors, in green and/or blue spaces was recognised as contributing to people's wellbeing, and offered mental health service users opportunities to feel restored, enjoy nature, be active and have social contact. It was mostly structured around errands or specific tasks and was considered to be an important part of a regular routine to help tackle negative aspects of their day. Spending time outdoors had the potential to elevate mood, ‘lift your spirits, lifts your mind’, helped to reduce unhelpful behaviours—whether these related to challenging feelings of fear, social anxiety or avoiding unhealthy eating habits. A clear link between exercise and wellbeing was understood and the concept that nature was restorative, soothing and clean and required protection and preservation was depicted by those interviewed. However, a range of practical and psychological barriers prevented access was evident. Lack of transport, physical health problems and feeling tired restricted some people's activities but having some social support (for the most part, provided by Praxis Care) to encourage them to leave the house was a valuable resource. Reflecting on the quantitative results, people without experience of mental health problems were more likely to have a higher frequency and dose of green and blue spaces compared to those with mental health problems. Unpicking why these differences were found is complicated however some light may be shed on the variation in motivations to spend time outdoors between the two groups. There were clear differences in how and why people accessed green and blue spaces, with the general sample more likely to endorse positively framed reasons such as getting exercise, enjoy the weather or gardening. Those without mental health problems endorsed items that focused on self‐improvement such as increasing physical activity and physical health, enjoying nature and the fresh air and investing time in themselves. Green and blue spaces were considered a way to improve both physical and mental wellbeing. Those with experience of mental health problems portrayed a more negative personal perspective of the barriers they faced. These included issues around self‐image, managing anxiety, feeling guilt or dealing with stress. Relying on external motivators was more likely to be endorsed such as walking a pet and having someone to encourage them to get outdoors. There were no differences between the two groups in a number of aspects including taking part in planned outdoor activities such as walking groups and outdoor gyms. Spending time outdoors to improve concentration, and motivation or to work through a problem was also similar. Meeting up with friends and family was equally important to both groups.

Going out at night was preferred by those with mental health problems by either measure. Further research could help explore the reasons why this might be the case and hypotheses could include the role of social anxiety, personal safety and disrupted circadian rhythms. Feeling safe at night is a particular issue for many women, creating safer spaces and greater awareness of the vulnerabilities women experience on a regular basis, better lighting and more effective outdoor security measures may help increase feelings of safety for everyone.

Like the participants in our study, the general public value the importance and benefits of green and blue spaces for wellbeing.^28^ Given the imminent pressures of climate change, green and blue spaces can play a role in helping mitigate some of the environmental challenges of climate change^65^, ^66^ and integrate the central role that public and private access to green and blue spaces has in public mental health promotion.^67^, ^68^ We face sustainability challenges, that require innovative and bold decision‐making to transform urban and rural planning, incentivise environmentally sensitive building design and provide outdoor recreation and safe spaces for social activity.^69^, ^70^, ^71^, ^72^ Too often, poor housing stock in deprived areas serves mental health service users in residential settings, with limited access to quality green and blue spaces.^65^ Our research would suggest that the role that social support plays in encouraging people to spend time outdoors is key, particularly for those working within social care that has the right skills and experience to support mental health service users to be more active. This approach should become integrated into treatment and care plans. The important role that social care providers offer can be reinforced by family members and friends who can make a difference by encouraging people to be active. Peer or family‐led interventions are an effective way to engage service users in behaviour change and these could be extended to include nature‐based green and blue approaches. Complementary strategies that could potentially reduce reliance on medication to help manage stress, anxiety and sleep could be promoted through green prescribing schemes and become mainstream approaches within mental health services.

Messaging about the benefits of green and blue spaces is equally important for people with mental health problems and must tackle the anxieties and concerns people raised, people should feel safe, secure and supported to access outdoors and be aware of the mental health benefits that may be available. Although our data did not highlight demographic differences in people's experiences of feeling unsafe, it is important to acknowledge, explore and consider that people may feel unsafe for a wide range of intersecting reasons, for example, in relation to gender, age, sexual orientation, physical appearance and being from visible minorities. Improving access to green and blue spaces has the potential to help address these inequities and the wider social determinants of health, but these complexities need to be considered in the development and promotion of safe, open and accessible places for everyone. How to create and promote accessible spaces for all should be a priority for planners and policymakers, and involving service users and carers in these processes will help to ensure all the relevant and complex issues are considered.

### 4.1 Implications for policy and practice

It is clear that there are different barriers and facilitators for people who are experiencing mental health problems. The emerging evidence on the contribution that green and social prescribing can make to reduce mental health inequalities is encouraging. Mental health providers, GPs, social workers, education and workplace settings should be encouraged to respond to and incorporate the research learning to establish opportunities and experiences to engage with nature in recognition of its therapeutic benefits. The development of policy guidance would be a welcome starting point and would acknowledge the need to adopt and embed sustainable practices. Inequalities extend to access to green and blue spaces and planning policies already address the need for improving access but this does not tackle existing estate and the fact that social housing stock is often located in areas of social deprivation. Interventions that promote feelings of safety, and reduce anxiety via buddy systems/could promote the social gateway to green and blue spaces. Promoting the benefits of being outdoors should use appropriate and relevant language and identify goals for those experiencing mental health problems such as improving self‐image, reducing anxiety, increasing social contact and enhancing mood. Improving access to green and blue spaces is a joint responsibility across government departments and policy needs to reflect this. The longer‐term impact of COVID‐19 on mental health is also an area of developing concern and we need to understand whether the impact of isolation has reinforced anxieties about being in now less familiar, crowded public spaces.

### 4.2 Strengths and limitations

We were unable to access an SMI sample because of Covid restrictions. To account for this deficit, we contacted service users living in supported living settings despite our directed efforts to recruit an SMI population, analyses found that almost half of the online participants reported mental health problems, including SMI. The majority of participants in the qualitative study were older adults therefore we cannot generalise these findings to younger people. Using MTurk to access a general population that included a large number of US participants and people were paid a small amount to participate, it is unlikely that participants were a random sample and therefore we are unable to generalise any findings to the United Kingdom.

## 5 CONCLUSION

Promoting green and blue spaces as a mental health prevention, early intervention or treatment option could be a valuable public mental health approach, but it is important that access to safe spaces is improved through public planning and policy. Ways of providing access, on whatever scale, within the existing estate of supported housing for people with mental health problems should be considered. The importance of appropriate messaging could help promote the appeal of green and blue spaces to different users. More research is required into the routine use of green and blue spaces and the potential positive impact on wellbeing.

## AUTHOR CONTRIBUTIONS

Claire McCartan and Gavin Davidson led all aspects of the project. Lee Knifton, Paul Webb and Chris White contributed to the study design and, with Liam Bradley and Katherine Greer, contributed to writing the research proposal and applying for and securing project funding. Liam Bradley, Katherine Greer and Paul Webb developed the qualitative interview schedule and Paul Webb facilitated data collection. Katherine Greer and Aodán Mulholland conducted the qualitative interviews. All authors contributed to the analysis and study write‐up. Each author certifies that they have made a direct and substantial contribution to the conception, design and delivery of the study. We are grateful to the service users who participated in the interviews and thanks to Emily Peckham, Piran White, Simon Gilbody and the Closing the Gap team for their support and guidance delivering the project.

## CONFLICT OF INTEREST STATEMENT

Gavin Davidson's post at Queen's University Belfast is partly funded by Praxis Care which was one of the partners in this project. The remaining authors declare no conflict of interest.

## ETHICS STATEMENT

Ethical approval for the qualitative stage was granted by the Research Ethics Committee of the School of Social Sciences, Education and Social Work at Queen's University Belfast in April 2019. The quantitative stage was approved by the NHS West Midlands—Coventry and Warwickshire Research Ethics Committee in July 2020 (REC reference 20/WM/0172). All participants provided written informed consent before taking part.

## Data Availability

Data are available on request.
